# Moss stable isotopes (carbon-13, oxygen-18) and testate amoebae reflect environmental inputs and microclimate along a latitudinal gradient on the Antarctic Peninsula

**DOI:** 10.1007/s00442-016-3608-3

**Published:** 2016-03-22

**Authors:** Jessica Royles, Matthew J. Amesbury, Thomas P. Roland, Glyn D. Jones, Peter Convey, Howard Griffiths, Dominic A. Hodgson, Dan J. Charman

**Affiliations:** British Antarctic Survey, High Cross, Madingley Road, Cambridge, CB3 0ET UK; Geography, College of Life and Environmental Sciences, University of Exeter, Exeter, EX4 4RJ UK; Department of Plant Sciences, University of Cambridge, Downing Street, Cambridge, CB2 3EA UK

**Keywords:** Climate change, Precipitation, Water line, Assemblage, Microtopography

## Abstract

The stable isotope compositions of moss tissue water (δ^2^H and δ^18^O) and cellulose (δ^13^C and δ^18^O), and testate amoebae populations were sampled from 61 contemporary surface samples along a 600-km latitudinal gradient of the Antarctic Peninsula (AP) to provide a spatial record of environmental change. The isotopic composition of moss tissue water represented an annually integrated precipitation signal with the expected isotopic depletion with increasing latitude. There was a weak, but significant, relationship between cellulose δ^18^O and latitude, with predicted source water inputs isotopically enriched compared to measured precipitation. Cellulose δ^13^C values were dependent on moss species and water content, and may reflect site exposure to strong winds. Testate amoebae assemblages were characterised by low concentrations and taxonomic diversity, with *Corythion dubium* and *Microcorycia radiata* types the most cosmopolitan taxa. The similarity between the intra- and inter-site ranges measured in all proxies suggests that microclimate and micro-topographical conditions around the moss surface were important determinants of proxy values. Isotope and testate amoebae analyses have proven value as palaeoclimatic, temporal proxies of climate change, whereas this study demonstrates that variations in isotopic and amoeboid proxies between microsites can be beyond the bounds of the current spatial variability in AP climate.

**Electronic supplementary material:**

The online version of this article (doi:10.1007/s00442-016-3608-3) contains supplementary material, which is available to authorized users.

## Introduction

The Antarctic Peninsula (AP) has experienced significant climate change over the past 50 years, with increasing temperatures and changing precipitation patterns (Turner et al. 2005, 2009, 2014). The area of ice-free land has increased (Arigony-Neto et al. 2009; Cook et al. 2005), the depth and continuity of permafrost have altered (Bockheim et al. 2013) and the melt season has lengthened (Barrand et al. 2013). Consequently, the microhabitats of native terrestrial flora and fauna have changed (Parnikoza et al. 2009; Royles et al. 2013a). An equally dynamic future is predicted, with increased potential for successful establishment by non-native species, alongside expansion of native populations (Chown et al. 2012; Convey 2011; Larsen et al. 2014).

Little is known about the responses of terrestrial biological systems to climate change during the short summer growing season (Convey 2010). Moss is widespread across ice-free areas of the AP and occasional deep moss banks provide rare terrestrial biological archives of environmental changes, spanning timescales up to 5000 years (Björck et al. 1991; Royles et al. 2012). Peat accumulation rates, mineral composition and extent of humification have been interpreted as temperature- and moisture-driven environmental proxies (Björck et al. 1991; Fenton 1980). More recently, the stable carbon (C) isotope composition (δ^13^C) of cellulose has been used as a proxy for the carbon dioxide (CO_2_) assimilation rate (Royles et al. 2012), whilst testate amoebae provide an indication of microbial activity (Royles et al. 2013a).

Ombrotrophic mosses are dependent on precipitation and the isotopic composition of this source water (δ^18^O_SW_) is an important determinant of moss cellulose composition (δ^18^O_C_) (Barbour 2007). Cellulose is the major degradation-resistant component of bryophyte organic matter and is therefore a potential palaeoclimate archive. Understanding the relationship between δ^18^O_SW_ and the composition of leaf water (δ^18^O_L_) during cellulose synthesis is essential for reconstructing δ^18^O_SW_ from preserved cellulose as δ^18^O_L_ is frequently isotopically enriched compared to δ^18^O_SW_ (Barbour 2007). Being poikilohydric, moss water content can be highly variable over short time periods, although mosses may be metabolically active only during a narrow range of conditions. Thus, δ^18^O_L_ at most reflects the isotopic composition of precipitation between two dry periods and is essentially a short-term indicator, whilst δ^18^O_C_ integrates ^18^O inputs and exchanges throughout the period of growth.

For mosses, external water is a critical component of metabolism as it limits the CO_2_ diffusion rate from atmosphere to chloroplast. Photosynthetic CO_2_ assimilation and associated C isotope discrimination profiles initially increase as external water evaporates and then decline as tissues subsequently desiccate and metabolism ceases (Rice and Giles 1996; Royles et al. 2013b; Williams and Flanagan 1996). As a proportion of assimilated C is used to synthesise cellulose, the C isotope ratio of moss cellulose (δ^13^C_C_) is a good proxy for conditions during photosynthesis (Royles et al. 2012).

Changes in testate amoebae populations are widely used as a palaeohydrological proxy in temperate peatlands (Mitchell et al. 2008) and the proxy has recently been applied to the AP (Royles et al. 2013a). Temperature, moisture, pH and biogeographical factors all influence the contemporary distribution and population dynamics of moss-dwelling testate amoeba around Antarctica (e.g. Mieczan and Adamczuk 2014; Smith 1992, 1996; Todorov and Golemansky 1999). Populations are dominated by small, cosmopolitan taxa such as *Corythion*, *Trinema* and *Euglypha* spp., with diversity generally lower than in temperate regions (Smith 1992). Testate amoebae are dominant heterotrophic organisms in peatlands (Gilbert et al. 1998; Jassey et al. 2013; Mitchell et al. 2003) so their biomass and concentration can be used to assess microbial community responses to external drivers (Ju et al. 2014; Mitchell 2004; Payne and Mitchell 2009). Rapid population growth was coincident with recent AP climate change, suggesting that temperature, through its influence on food availability and reproduction rate, is a primary driver of microbial activity (e.g. Royles et al. 2013a). However, previous research on the effect of temperature on testate amoeba in temperate and sub-Arctic peatlands is uncertain and contradictory (Jassey et al. 2013; Payne 2014; Tsyganov et al. 2012).

Ecosystem responses over time, at two trophic levels, to AP climate change can be detected using multi-proxy analysis (peat accumulation rate, δ^13^C, testate amoebae) of moss–peat cores (Royles et al. 2013a). Here, using contemporary *Chorisodontium aciphyllum* and *Polytrichum strictum* surface samples from four sites over a >600-km latitudinal gradient, we measure the modern analogues of these palaeoclimate proxies (δ^13^C_C,_ δ^18^O_C_, moss tissue water δ^18^O and δ^2^H, testate amoebae assemblages). Our primary aim is to test these against measurements of precipitation stable isotope composition, tissue water content and (micro-)climate in order to investigate the response of moss and amoebae to environmental and climate gradients, to test their applicability in palaeoclimate studies. This provides vital context to understand the contemporary processes driving the proxy signals, which are preserved over thousands of years in the moss bank core samples. Our hypotheses are that:

1. Moss water and cellulose δ^18^O values are recorders of precipitation isotope ratios.
2. Cellulose δ^13^C is a record of photosynthetic assimilation and growing season length with lower latitude sites discriminating more against ^13^C.
3. Across the latitudinal gradient, testate amoeba community composition diversifies, and concentration and biomass increase, as a result of higher temperatures and/or increases in water availability.

## Materials and methods

### Sample sites

**Fig. 1:**
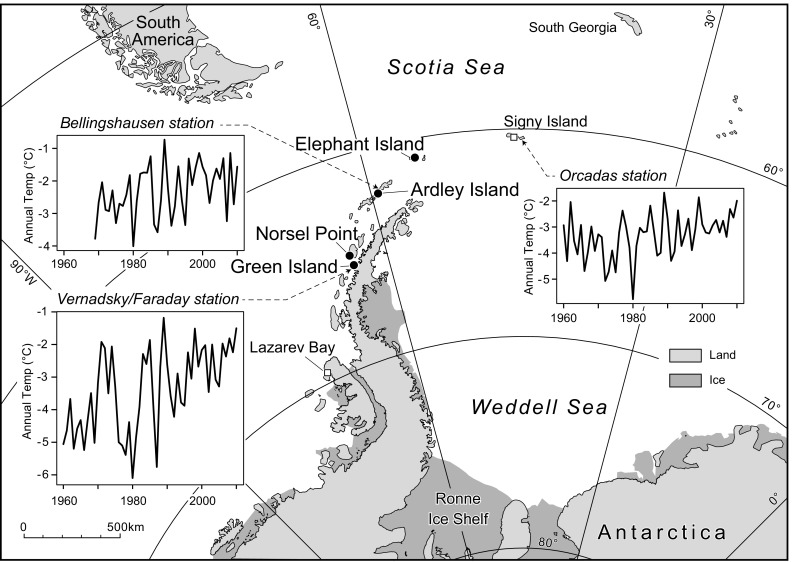
Map of Antarctic Peninsula (AP) region with new sample sites marked (*black circles*). Other relevant sites are shown (*white squares*).* Inset* graphs show mean annual temperature records since 1960 from Orcadas (South Orkney Islands), Bellingshausen (South Shetland Islands) and Vernadsky/Faraday (Argentine Islands) stations

**Fig. 2:**
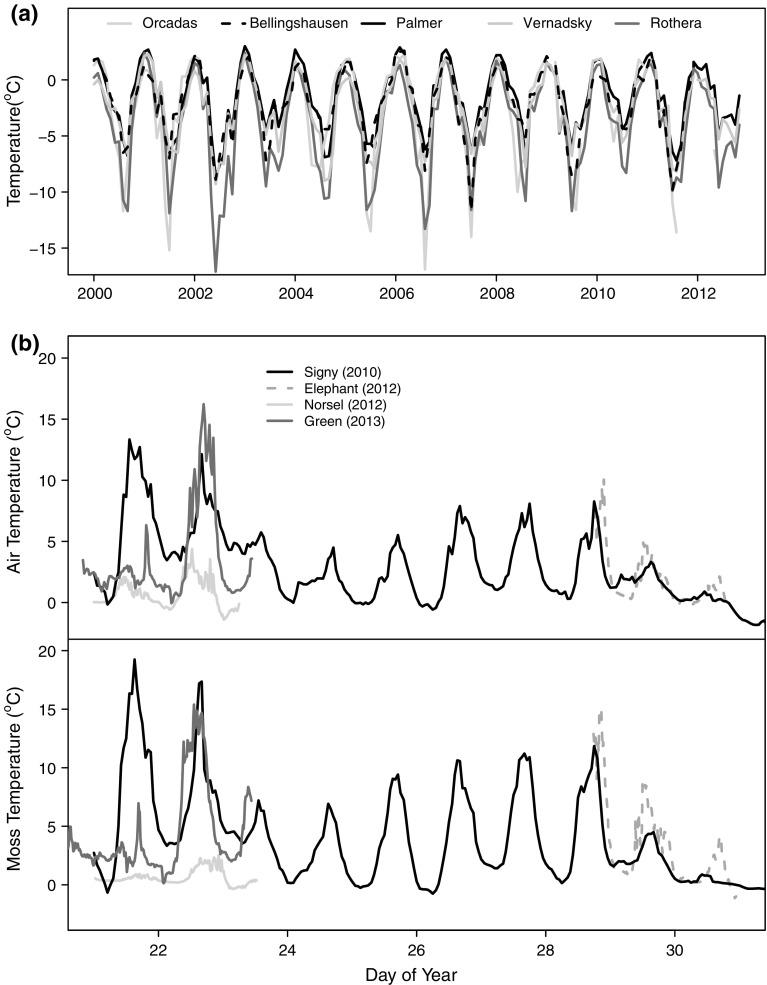
**a** Mean monthly air temperatures recorded at AP meteorological stations (Scientific Committee on Antarctic Research 2014); **b** short-term temperature measurements at moss surface and non-protected air temperature above the moss at Elephant, Norsel and Green Island, compared with daily readings made on Signy Island in 2009–2010

During January 2012 and January 2013, sixty-one sites on the western AP, divided between Green Island (GRE), Norsel Point (NOR), Ardley Island (ARD) and Elephant Island (ELE) (Fig. 1; Table S1) were visited. Surface moss samples (5 cm × 5 cm × 5 cm), water samples (>1 ml, snow banks, fresh precipitation, water pools) and short-term temperature measurements (Fig. 2) were collected. Water samples were also collected opportunistically from other AP locations (Table S2). Moss tissue and water samples were stored frozen until analysis. Tissue moisture content [(fresh mass − dry mass)/dry mass] and bulk density (Table S1) were determined by excising ca. 1-cm^3^ cuboids from each frozen moss sample which were precisely measured and dried to a constant mass.

### Isotope analyses of water and cellulose

δ^18^O and δ^2^H isotope ratios of source waters were determined using cavity ring-down spectroscopy (Lis et al. 2008) at the Centre for Stable Isotope Biogeochemistry, University of California, Berkeley (CSIB). Long-term precision for δ^2^H is ±1.0 ‰ and for δ^18^O is ±0.14 ‰.

Internal and external moss tissue water (collectively called “moss water”) was extracted by cryogenic vacuum distillation (Ehleringer et al. 2000; West et al. 2006). ^2^H/^1^H of the moss water was analysed at CSIB, using a Thermo H/Device interfaced with a Thermo Delta Plus XL mass spectrometer. Moss water ^18^O/^16^O was analysed using two methods. Samples >1 ml were analysed at CSIB using a Thermo Gas Bench II interfaced to a Thermo Delta Plus XL mass spectrometer (precision ±0.12 ‰). Low-volume samples were analysed following equilibration with CO_2_, using an isotope ratio mass spectrometer (SIRA; Isotech). Measured δ^18^O values were normalised relative to Vienna Standard Mean Ocean Water (VSMOW) using known standards. Samples analysed in both laboratories returned values that did not differ significantly.

Cellulose was extracted from 0.2-g dry mass moss samples following Loader et al. (1997), but replacing the 17 % sodium hydroxide step with a final 1-h bleaching (14 g sodium chlorite, 10 ml glacial acetic acid, 1 l deionised water) in a 70 °C water bath. For δ^13^C_C_ analysis, 1-mg samples of dry cellulose were transferred into tin capsules and measured at the Natural Environment Research Council (NERC) Isotope Geosciences Laboratory (British Geological Survey) by combustion in a furnace (Carlo Erba NA1500) connected to a dual-inlet Isotope-ratio mass spectrometer (VG Triple Trap and Optima). Sample ^13^C/^12^C isotope ratios were referenced to the Vienna Pee Dee belemnite scale using a laboratory standard calibrated against NBS-19 and NBS-22. Replicate analyses indicated a precision of ±<0.1 ‰ (1 σ).

For δ^18^O_C_ analysis, 1-mg samples of dry cellulose were transferred into silver capsules and analysed at Godwin Laboratory, University of Cambridge using a Thermo Finnigan 253 stable isotope ratio mass spectrometer with a high temperature conversion elemental analyser (Thermo Fisher Scientific). Two laboratory standards, and an international standard, NBS 127, were used to calibrate the reference gas. ISODAT software was used to calculate the sample values relative to this reference gas and anchor δ^18^O_C_ to the VSMOW scale.

Statistical analyses were performed using R version 3.0.2 (R Core Development Team 2014). To determine significant pairwise comparisons, normality of data was tested and either one-way ANOVA or Kruskal–Wallis rank sum tests applied, followed by post hoc Tukey tests or multiple comparison tests after Kruskal–Wallis [kruskalmc in the pgirmess package (Giraudoux 2014)] as appropriate.

### Testate amoebae populations

To isolate testate amoebae, 2-cm^3^ organic matter samples were prepared using standard techniques (Booth et al. 2010; Charman et al. 2000), with counts completed on the 300- to 15-μm fraction. Concentration of tests per cubic centimetre was calculated from the addition of an exotic spore tablet [*Lycopodium* (Stockmarr 1971)] and converted to concentration of tests per dry gram using bulk density values (Table S1). Minimum counts of 100 tests per sample were sought but total counts of 50–99 (*n* = 21) and 25–49 (*n* = 10) were accepted. Although these totals are lower than generally recommended (Payne and Mitchell 2009), they provide robust concentration data and reasonable estimates of assemblage composition given the low overall taxonomic diversity. In two samples it was not possible to obtain a count ≥25 and these were excluded.

Testate amoeba biomass estimates were calculated using standard techniques (Gilbert et al. 1998; Ju et al. 2014; Mitchell 2004; Payne et al. 2010), assuming geometric test shapes (Mitchell 2004), with dimensions applied based on microscopic measurements of shells (Table S4). Shell volumes were converted to C biomass using the factor 1 μm^3^ = 1.1 × 10^−7^ μg C (cf. Mitchell 2004; Weisse et al. 1990).

Shannon Wiener diversity index (SWDI) values (Sageman and Bina 1997) were calculated for each sample using the following equation:

$${\text{SWDI}} = - \mathop \sum \limits_{i = 1}^{S} \left( {{\raise0.7ex\hbox{${X_{i} }$} \!\mathord{\left/ {\vphantom {{X_{i} } {N_{i} }}}\right.\kern-0pt} \!\lower0.7ex\hbox{${N_{i} }$}}} \right) \times \ln \left( {\frac{{X_{i} }}{{N_{i} }}} \right)$$

where *X*_*i*_ is the abundance of each taxon, *N*_*i*_ is the total abundance of the sample, and *S* is the sample species richness. SWDI values of 0 (*n* = 5) were generated for mono-specific samples.

Cluster analysis of testate amoeba data was completed using the cluster package (Maechler et al. 2014) in R version 3.0.2 (R Core Development Team 2014). An agglomerative hierarchical method was applied (function hclust) with a Bray-Curtis distance measure, Ward’s linkage method and no standardisation of variables (cf. Swindles et al. 2009). Primary cluster groups (i.e. 1, 2) were defined at a set level of similarity (distance = 1.5), with subgroups (i.e. 2A, 2B) assigned using the natural grouping of the dendrogram (Fig. S1). Cophenetic correlation, which calculates the correlation between the original distance matrix and the dendrogram, was used to assess the viability of the assigned clusters.

The relationship between the testate amoeba community (with proportions of taxa recorded as concentration of tests per dry gram) and environmental variables (latitude, moisture content, altitude and aspect) was investigated using canonical correspondence analysis (CCA) ordination in Canoco version 4.5 and CanoDraw version 4 (ter Braak and Šmilauer 2002). *Euglypha rotunda*, *Euglypha tuberculata* type, *Hyalosphenia elegans* and *Hyalosphenia* sp. were excluded as rare species. Vegetation data (Table S1) were included as passive variables, as host moss type can be inter-correlated with other environmental variables and may not assert direct control on the testate amoeba assemblage (cf. Swindles et al. 2009).

## Results

### Contemporary measurements of climate and isotopic environment

#### Meteorological and microclimate conditions

Mean monthly air temperatures recorded at Bellingshausen (62°12′S 58°58′W, near ARD) and Vernadsky stations (64°14′S 64°15′W, near GRE) between 2000 and 2012 fall in the range of −9 °C to +2 °C, whilst annual temperatures remain below 0 °C, but show warming trends (Figs. 1, 2; Scientific Committee on Antarctic Research 2014).

Microclimate data were recorded for only a few summer days, but moss surface and overhead air temperatures were tightly coupled, without temporal lag (Fig. 2b). Daily minimum temperatures were approximately 0 °C, whilst the variation in daily maximum temperatures at a given site (e.g. approximately 10 °C range in maximum temperature measured at ELE) were similar to the range measured across the latitudinal gradient. The short-term measurements from ELE, NOR and GRE fit well with the longer term microclimate data recorded at Signy Island in 2009–10 (Fig. 2; Royles 2012). Moss temperatures were generally slightly warmer than air temperatures, but both reached maxima (ca. 15 °C) substantially higher than the mean monthly temperatures due to direct radiative processes.

#### Species composition and physical characteristics of moss

Moss samples comprised *C. aciphyllum* and *P. strictum*, the two known bank-forming species in this region (Table S1). Moisture content of the tissue at collection varied between 39 and 85 %, but the inter-quartile range (IQR) was narrow, 69–75 % (Table S1). The bulk density ranged from 0.052 to 0.197 g cm^−3^, with an IQR of 0.09–0.12 g cm^−3^ (Table S1).

#### Isotopic composition of contemporary source water

**Fig. 3:**
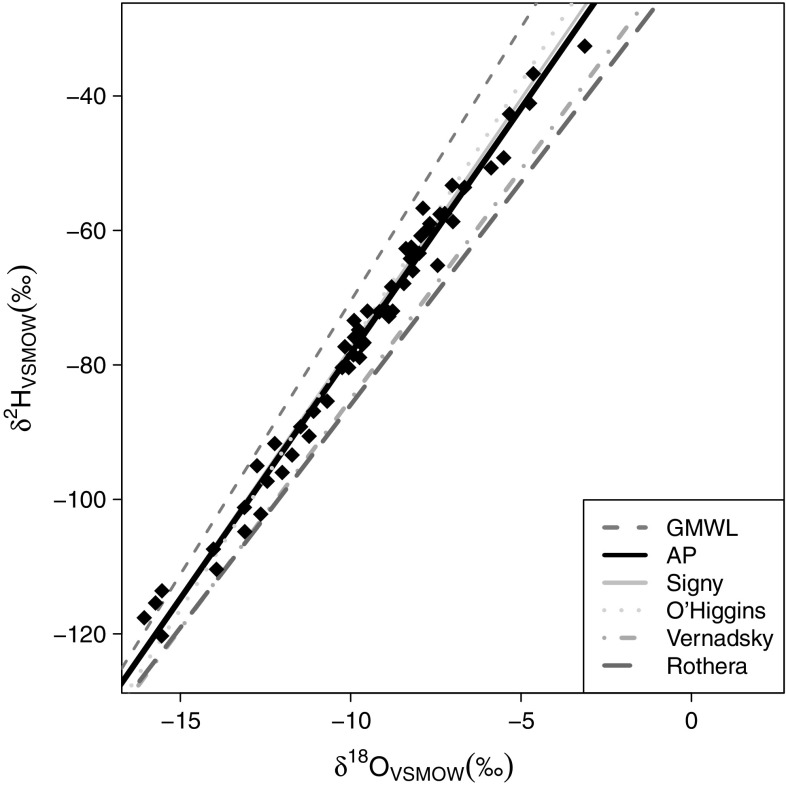
AP local meteoric water line (*LMWL*) generated from fieldwork samples (*black diamonds* and *line*), compared with LMWL from O’Higgins station (Fernandoy et al. 2012), Signy Island (Royles et al. 2013c), and International Atomic Energy Agency Global Network of Isotopes in Precipitation data from Vernadsky and Rothera. Highly significant linear model fitted to 57 water samples, equation of LMWL: *y* = 7.3*x* − 5.4, *r* ^2^ = 0.99, *P* < 0.0001. *VSMOW* Vienna Standard Mean Ocean Water

The isotopic composition of AP precipitation, including all 57 rain and snow samples, was well explained by a highly significant local meteoric water line (LMWL; δ^2^H as a function of δ^18^O) with a gradient of 7.3, below that of the global meteoric water line (GMWL; δ^2^H = 8δ^18^O + 10) across the measured range of δ^18^O values (−2 to −16 ‰; Fig. 3). The AP LMWL lies above those from Rothera and Vernadsky/Faraday, the closest International Atomic Energy Agency (IAEA) Global Network of Isotopes in Precipitation (GNIP) stations, but is similar to LMWLs for Signy Island (Royles et al. 2013c) and O’Higgins station (Fernandoy et al. 2012), which were also derived from summer precipitation collections.

#### Isotopic composition of moss water

**Fig. 4:**
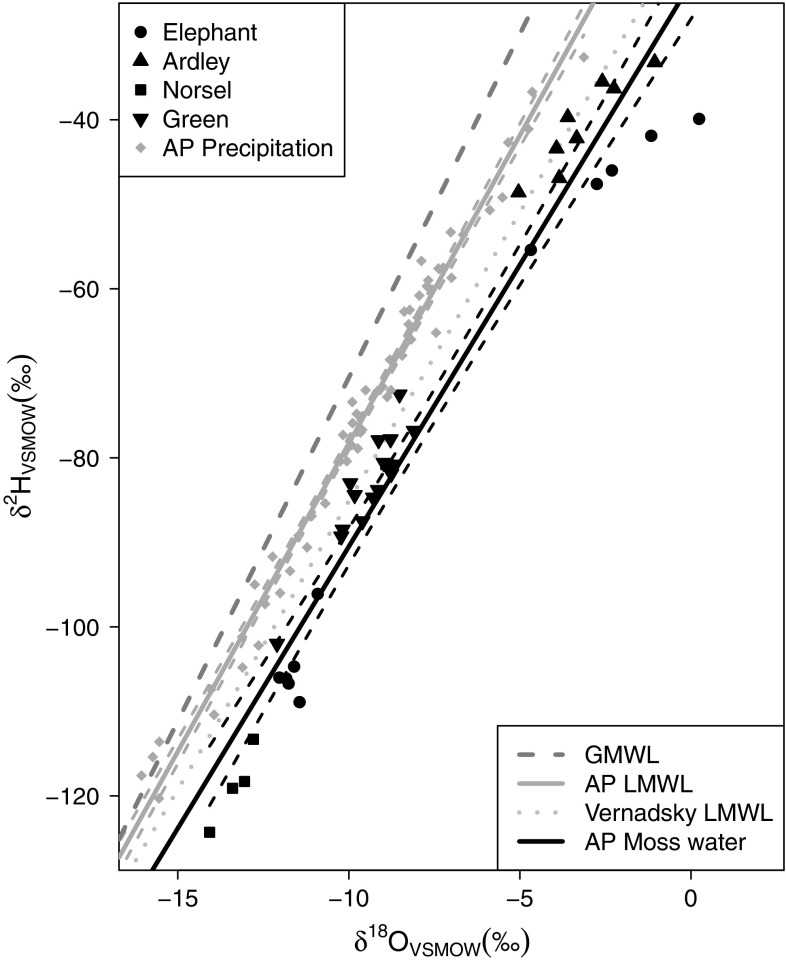
Water lines (δ^2^H vs. δ^18^O) for AP source water samples (*light grey* *y* = 7.3*x* − 5.4, *r* ^2^ = 0.99, *n* = 57, *P* < 0.0001) and moss water samples (*black* *y* = 6.7*x* − 23.8, *r* ^2^ = 0.95, *P* < 0.0001) in which water was distilled from surface moss samples. *Long dashed line* represents the global meteoric water line (*GMWL*; *y* = 8*x* − 10), *dotted line* is Vernadsky LMWL. *Dashed lines* represent 95 % confidence intervals of linear models

The moss water samples (Fig. 4) fell along a highly significant water line (*R*^2^ = 0.95, *p* < 0.0001), with a similar range in δ^18^O values but a lower gradient (6.9) than the LMWL (7.3). The samples were generally geographically clustered, with samples from ARD and ELE (sub-site 1) at the relatively enriched end between 0 and −5 ‰, the samples from GRE dominating the −5 to −10 ‰ zone, and NOR samples more negative than −12 ‰. A second cluster of samples from ELE (sub-sites 2 and 3) had δ^18^O values of approximately −10 ‰. The moss water samples reflect a combination of cellular constituents and external interstitial waters surrounding the living tissues, but fall strikingly close to the LMWL for Vernadsky, the local GNIP station (Fig. 4). In addition to recent precipitation inputs, other contributory factors would be winter snowmelt, the extent of thaw and freeze thaw cycles, and exchanges with atmospheric water vapour. The divergence from the LMWL in a subset of more enriched moss water samples from ELE and ARD is not considered to be significant in terms of evaporative recycling, but perhaps reflects repeated freeze-thawing cycles, as freezing affects δ^18^O and δ^2^H values differently, decreasing the slope of the MWL.

**Fig. 5:**
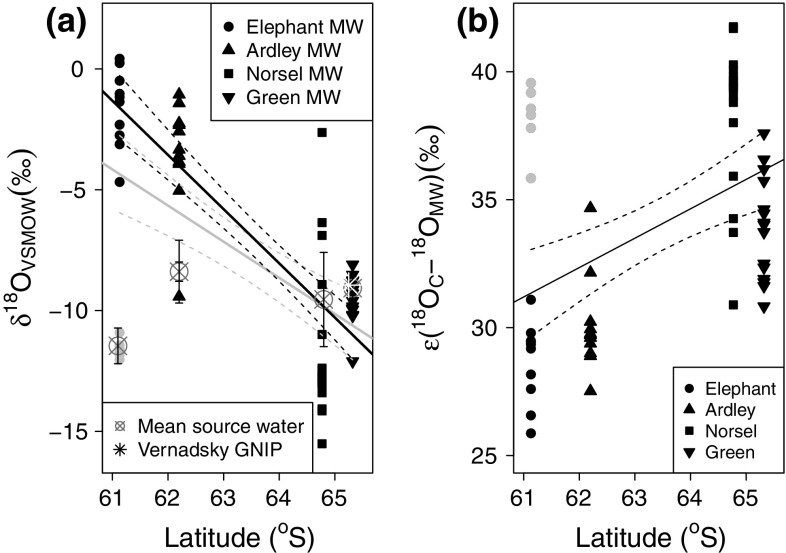
**a** δ^18^O composition of moss water (*MW*) as a function of latitude. *Dots* Elephant Island (*ELE*; *l* *ight grey dots* represent data from ELE sites 2 and 3), *upward triangles* Ardley Island (*ARD*), *squares* Norsel Point (*NOR*), *downward triangles* Green Island (*GRE*). *Grey line* significant linear model fitted to all data, plotted with 95 % confidence interval; *black line* significant linear model fitted to* black data points* (excluding ELE sites 2, 3) plotted with 95 % confidence interval. *Grey cross-hairs* represent mean δ^18^O composition of source water (*error bars* represent SE), *white asterisk* indicates January precipitation at Vernadsky. **b** Enrichment of δ^18^O_C_ relative to δ^18^O_moss-water_ as a function of latitude. *Black line* significant linear model fitted to all data (*P* = 0.0011, *r* ^2^ = 0.14) and plotted with 95 % confidence interval. *Light-grey dots* represent data from ELE sites 2 and 3

The moss water δ^18^O (δ^18^O_MW_) values were significantly dependent upon latitude (Fig. 5a), although the range of values measured at each location (over 10 ‰) exceeded the 7 ‰ variation in the linear model fitted across the latitudinal range. The bimodal distribution of the ELE moss water δ^18^O values is in contrast with the continuous distributions of values from the other locations. Whilst a highly significant linear model can be fitted when the ELE site 2 and 3 samples are included [Fig. 5a (grey line); *p* < 0.0001, *R*^2^ = 0.33], substantially more variation is explained by the linear model when those points are excluded [Fig. 5a (black line); *p* < 0.0001, *R*^2^ = 0.67]. At NOR and GRE mean δ^18^O_SW_ fell in the middle of the range of δ^18^O_MW_ values, at ARD δ^18^O_SW_ was at the depleted end of the δ^18^O_MW_ values, whilst at ELE, δ^18^O_SW_ was around −12 ‰, similar to that of the moss water from ELE sites 2 and 3, and around 10 ‰ more depleted than that of the ELE site 1 moss water. There was no significant relationship between δ^18^O_MW_ and δ^18^O_C_ from associated moss tissue within or between locations. The enrichment factor between cellulose and moss water ranges 26–42 ‰ and increases with latitude (Fig. 5b), though again the ELE sites demonstrate a bimodal distribution, driven by the more negative δ^18^O_MW_ samples from ELE 2 and 3.

### Measurements of modern proxy analogues

#### Isotopic composition of cellulose

**Fig. 6:**
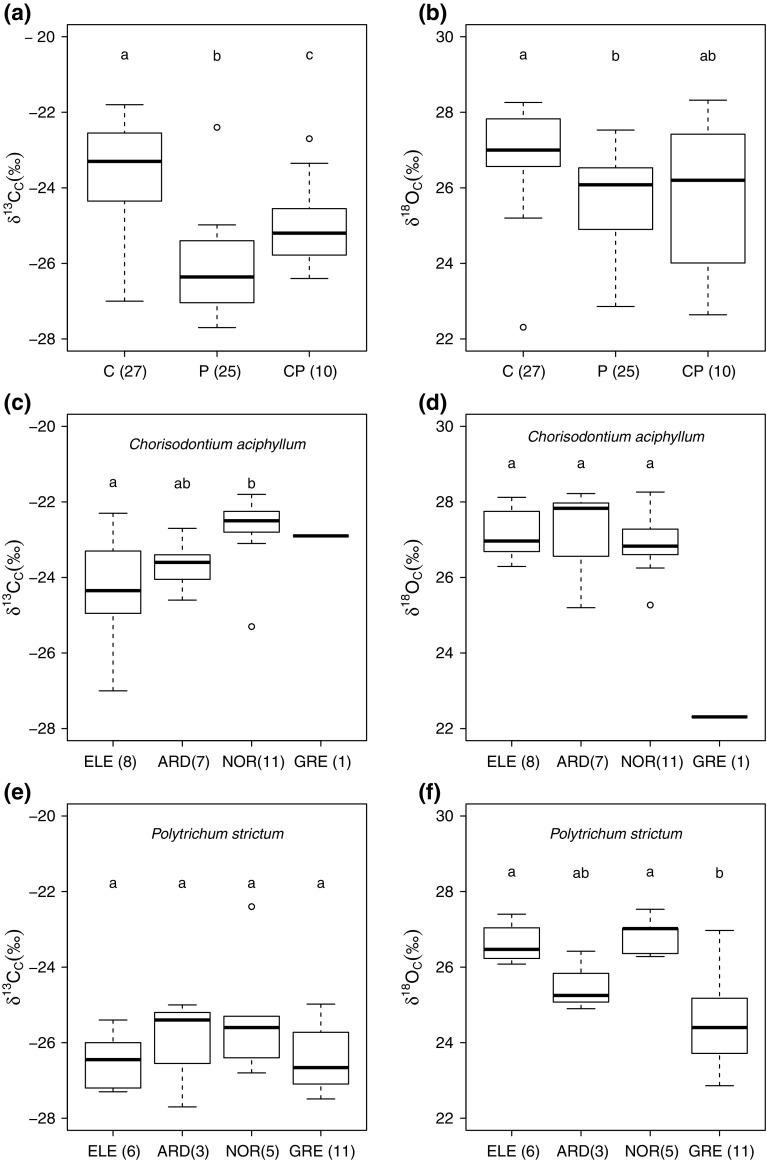
Stable isotope composition of cellulose for surface moss samples of bank-forming species (number of sites included); *different letters* denote significantly different groups. **a** δ^13^C and **b** δ^18^O composition of all *Chorisodontium aciphyllum*, *Polytrichum strictum* and mixed samples (*C. aciphyllum*/*P. strictum*); **c** δ^13^C and **d** δ^18^O composition of pure *C. aciphyllum* samples, divided by island; **e** δ^13^C and **f** δ^18^O composition of pure *P. strictum* samples, divided by island. *Boxes* represent inter-quartile range (IQR) of data, *whiskers* extend to a maximum of 1.5 IQR. For other abbreviations, see Fig. 5

With a mean 3 ‰ offset between species, δ^13^C_C_*P. strictum* was significantly more negative than δ^13^C_C_*C. aciphyllum* (Fig. 6a). Along with species, the moisture content of the moss tissue was a significant explanatory factor of the δ^13^C_C_ values, with less negative δ^13^C_C_ values associated with wetter tissue (ANOVA, species, *F* = 85.6, mean square error = 100.54, *P* < 0.01; moisture content, *F* = 7.56, mean square error = 8.87, *P* < 0.01). The δ^18^O composition of *C. aciphyllum* was significantly more enriched than that of *P. strictum* (Fig. 6b). The mixed species samples had isotopic compositions intermediate between the monospecific samples.

The δ^13^C_C_ values of ELE *C. aciphyllum* samples were significantly more negative than the NOR samples, but the other sites were indistinguishable (Fig. 6c) and there were no significant inter-site differences in *P. strictum* δ^13^C_C_ values (Fig. 6e). Whilst there were no significant differences in *C. aciphyllum*^18^O_C_ values between the islands, the single sample from GRE was 5 ‰ more depleted than the very consistent values of 27 ‰ measured from ELE, ARD and NOR (Fig. 6d). For *P. strictum*, the most southerly analysed samples, those from GRE were significantly more depleted than those from NOR and ELE (Fig. 6f).

#### Testate amoebae assemblages

**Fig. 7:**
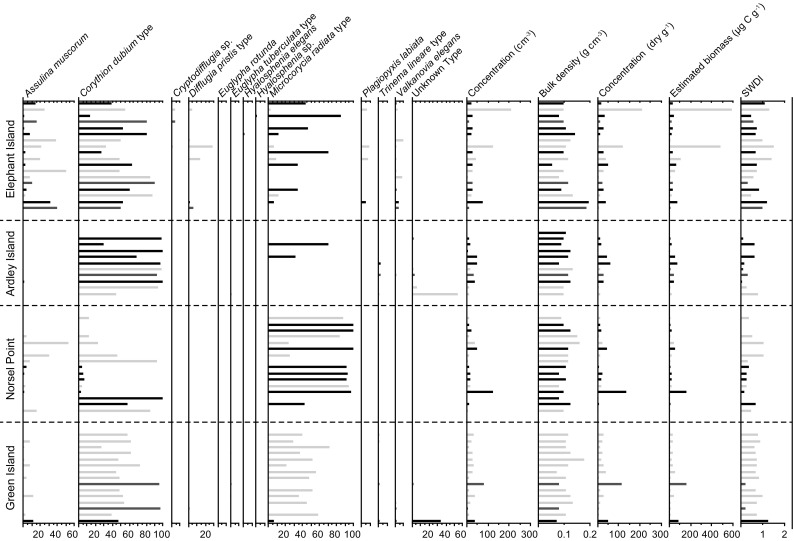
Testate amoebae distribution and abundance, sub-divided by site (Table 1) and colour coded by moss type (*light grey* *P. strictum*, *black* *C. aciphyllum*, *mid-grey* *C. aciphyllum*-*P. strictum* mix) (see “Testate amoebae populations” for details). *SWDI* Shannon Wiener diversity index

The testate amoebae assemblages had very low diversity (Fig. 7), with only 13 taxa identified, and eight of those occurring in fewer than ten samples with a maximum abundance <10 % (Table S3). *Corythion dubium* type was very widespread, occurring in 95 % of samples, the dominant taxon in 63 % of samples and highly morphologically variable with a mean length of 41 μm (range = 27–57 μm, 1* σ* = 7 μm) and width of 27 μm (range = 17–38 μm, 1* σ* = 5 μm). *C. dubium* was previously classified into size fractions (Royles et al. 2013a) but here we treat it as one class, until any systematic morphological and genetic differences that exist within the morphotype are determined (e.g. Bobrov et al. 1999; Oliverio et al. 2014). *Microcorycia radiata* type was identified in the Antarctic region for the first time (Meisterfeld, unpublished database), and occurred in 67 % of samples, whilst *Assulina muscorum* also occurred frequently (61 % of samples) but was less dominant than either *C. dubium* or *M. radiata* types (Table S3). Unknown type occurred in only seven samples (12 %), but was the dominant taxon in one ARD sample.

**Fig. 8:**
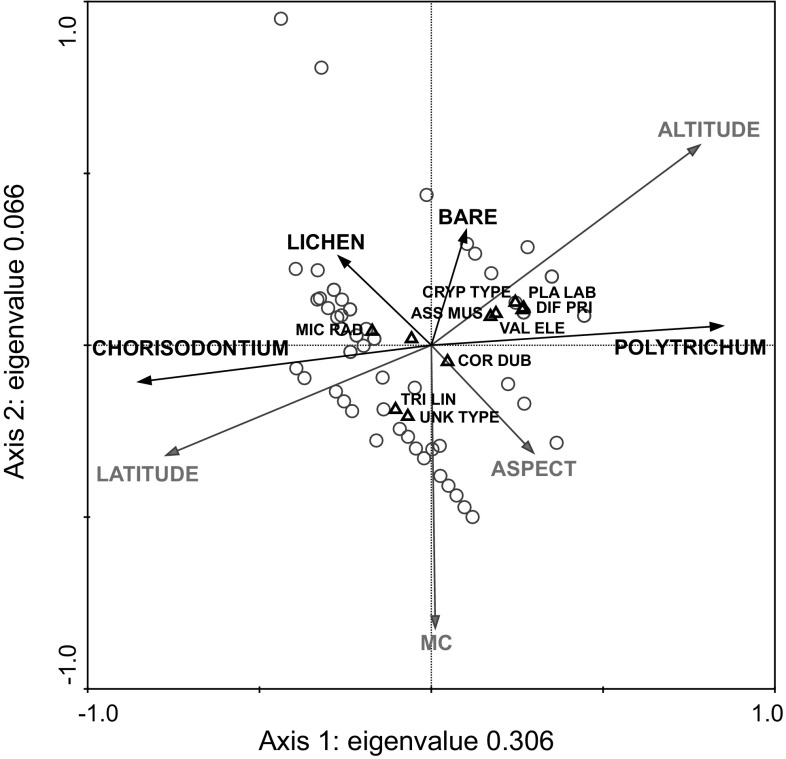
Results of canonical correspondence analysis showing environmental variables (*grey*), passive environmental variables (i.e. vegetation types; *black*), taxa (*triangles*; labelled) and samples (*grey circles*). *MC* Moisture content

Mean sample concentrations (27,830 tests dry g^−1^; Tables 1; S3) were less than half those reported from an Alaskan Arctic fen (Mitchell 2004), but had double the SD. Mean sample biomass (49.8 μg C g^−1^; Tables 1, S3) was considerably lower than that reported by Mitchell (2004), reflecting the dominance of small taxa. Regressions of biomass estimates against mean microclimate temperature (not shown) resulted in no significant correlations. Cluster analysis divided the testate amoebae dataset into four primary groups either dominated by *M. radiata* type (group 1) or *C. dubium* type (group 2), containing primarily a mixture of the two (group 4), or a more diverse assemblage (group 3; Fig. S1). A high cophenetic correlation (0.768) suggested that these clusters were a reliable representation of the original dataset. CCA axes 1 (eigenvalue 0.306) and 2 (eigenvalue 0.066) together explained 26.8 % of the variability in the testate amoeba data and 98 % of the species-environment relationship (Fig. 8; Table S5). Latitude and altitude were the environmental variables most strongly related to axis 1, with the two main moss taxa, *C.aciphyllum* and *P. strictum*, positioned at opposite ends of the axis reflecting the broad geographic pattern observed in the distribution of the two bank-forming species. Axis 2 was principally related to moss moisture content, which is an important determinant of testate amoeba assemblages in temperate peatlands (e.g. Amesbury et al. 2013; Swindles et al. 2009). However, our data do not support this explicit hydrological link. In line with the broadly cosmopolitan testate amoeba assemblage, most taxa clustered in the centre of the ordination space without clear alignment along either of the principle axes of variability. This was especially true of the dominant taxon *C. dubium* type, which fell almost at the 0, 0 intercept.

**Table 1:** Summary testate amoeba data by site

Site	Concentration (tests dry g^−1^)		Biomass (μg C g^−1^)		SWDI	
	Mean ± 1 SD	Median	Mean ± 1 SD	Median	Mean ± 1 SD	Median
Signy^a^	157,634 ± 254,898	73,404	912 ± 1943	121	1.18 ± 0.50	1.14
Elephant Island	38,521 ± 50,436	26,010	92 ± 164	34	0.86 ± 0.35	0.75
Ardley Island	19,897 ± 18,119	10,114	27 ± 23	13	0.30 ± 0.28	0.28
Norsel Point	21,001 ± 32,368	13,920	27 ± 39	21	0.36 ± 0.34	0.18
Green Island	26,253 ± 26,426	20,991	35 ± 39	28	0.73 ± 0.26	0.74
Lazarev Bay^b^	16,630	–	21.48	–	0.04	–
All sites	27,831 ± 35,907	16,935	49.8 ± 97.9	21	0.59 ± 0.39	0.64

*SWDI* Shannon-Wiener diversity index

^a^Unpublished data from Signy Island, South Orkney Islands

^b^Lazarev Bay, Alexander Island (Royles et al. 2013a); one data point included based on top 3.2 cm of core for which bulk density values were available to calculate concentration (tests dry g^−1^)

## Discussion

### Contemporary growth environment

Mean annual temperatures on the AP are sub-optimal for *C. aciphyllum* and *P. strictum* (Davey and Rothery 1997); however, moss surface temperatures are often substantially warmer than air temperatures (Fig. 2b) (e.g. Bramley-Alves et al. 2014; Smith 1988), with diurnal variation exceeding the latitudinal AP temperature cline. As temperature sensitivity responses are generally shallow, despite the sub-optimal air temperature, AP mosses can regularly photosynthesise at a high proportion of their maximal rate (Convey 1996; Longton 1988). As shown by the species compositions reported here, whilst *P. strictum* and *C. aciphyllum* are both found across the AP, *C. aciphyllum* is the dominant bank-forming species at northern sites, and *P. strictum* dominates the drier, southerly sites (Fenton and Smith 1982).

### Relationship between isotopic composition of source water and moss tissue water

The AP LMWL was almost identical to the Signy Island (Royles et al. 2013c) and O’Higgins station LMWLs (Fernandoy et al. 2012), which are located to the north-east and east of our sampling sites, respectively (Figs. 1, 3). Water samples were collected close to sea level, on windy, oceanic islands, so altitude or rain-out effects were minimal, as shown by the consistency of the LMWL across the sample transect. The moss water line is distinct from the LMWL (Fig. 4). The moss water is likely to represent water integrated over a longer time period than the precipitation samples, which contain water from a single precipitation event. Analysis of the GRE water samples shows this temporal integration and mixing of precipitation into the moss water. Whilst the GRE LMWL lies above the Faraday/Vernadsky LMWL, the GRE moss water line is very similar to the Faraday/Vernadsky LMWL (Fig. 4). In addition, the range of δ^18^O values contributing to the LMWL (6 ‰) exceeded that of the moss water (2 ‰). The mean monthly δ^18^O values of summer (December–March) precipitation measured at Vernadsky fall between −8.5 and −9 ‰ [IAEA and World Meteorological Organization (WMO) 2014] compared to moss water δ^18^O values of −8 to −10 ‰. Thus, in line with hypothesis 1, the isotopic composition of moss water provides a good approximation of summer precipitation composition, better than that obtained from spot collections of precipitation. Furthermore, the moss water oxygen isotope composition partially reflected the well-recognised latitudinal effect on the isotopic composition of precipitation (Dansgaard 1964; Gat 1998) (Fig. 5a); however, there was not a significant relationship between δ^18^O_SW_ and latitude. Sampling in remote AP locations was necessarily spatially and temporally limited, potentially confounding comparisons between locations. However, the linearity of the AP LMWL and moss water line, the samples not being collected in latitudinal order and the overlap in temperature measurements made across the transect give confidence to our cross-transect comparisons.

The approximate 10 ‰ separation at ELE between δ^18^O_MW_ values from sub-site 1 compared to sub-sites 2 and 3 is clear. The sites are within 1 km of each other, so the timing and isotopic composition of precipitation should be consistent. One explanation is that the very exposed sub-site 1, from a 3-m-deep moss bank, is solely fed by relatively isotopically enriched summer rain, as the slope and aspect of the bank are unable to support winter snow banks. In contrast, the banks at sub-sites 2 and 3 have lower profiles above the ground surface and shallower inclines and thus may have incorporated more isotopically depleted snowmelt water, either from snow settling on the moss, or run-off from surrounding areas. Indeed, the two most isotopically enriched samples from ELE fell below the moss water line (Fig. 4), possibly indicating humidity-dependent secondary evaporative effects on LMWL source waters. However, the cool, windy AP conditions with frequent precipitation are generally unfavourable for multiple evaporative recycling events. The relative humidity/vapour pressure at the moss surface is generally high, with a low vapour pressure gradient to the atmosphere, and the low air temperatures reduce evaporative demand. Net evaporation of water from the moss surface is likely to be low, but there may be exchange between moss water and water vapour, with the isotopic composition of the vapour being imprinted onto the moss water and, potentially, cellulose (Ellwood et al. 2011; Helliker and Griffiths 2007).

### Cellulose δ^18^O and δ^13^C as palaeoenvironmental proxies on the AP

Across all sites the moss was growing in exposed, windy locations, without canopy cover and with minimal local sources of respiratory CO_2_. Consequently, we assume that all the mosses were assimilating atmospheric CO_2,_ with the associated contemporary δ^13^C signature of approximately −8 ‰ (Keeling et al. 2008; Rubino et al. 2013). Radiocarbon dating techniques show that *C. aciphyllum* on Signy Island (60°45′S) has recently accumulated at up to 3.9 mm year^−1^ (Royles et al. 2012) whilst *P. strictum* on Alexander Island (69°22′S) has been accumulating at approximately 4 mm year^−1^ (Royles et al. 2013a). Consequently it is expected that the surface moss samples, with green growing tips of approximately 5 mm, represent a similar growth period at all locations, despite the difference in latitude and species (Royles and Griffiths 2014).

The δ^13^C_C_ values are significantly more negative in *P. strictum* than in *C. aciphyllum* and, furthermore, are significantly more negative in moss samples with a lower moisture content, due to variation in the biochemical fractionation and/or diffusion resistance to CO_2_ (Farquhar et al. 1989). Polytrichales (including *P. strictum*) have surface lamellae which facilitate CO_2_ diffusion (Proctor 2005) and enable the maintenance of high rates of photosynthesis when other mosses would become either CO_2_ limited due to the covering water film or desiccated. Thus, for mosses with the same external water layer, the rate of CO_2_ diffusion will be higher in *P. strictum* than *C. aciphyllum*, facilitating greater fractionation against ^13^CO_2_ (Rice and Giles 1996). In addition, a higher proportion of the seasonal net assimilation will occur under high discrimination conditions, which is reflected in a more negative seasonally integrated δ^13^C_C_ value (Royles et al. 2012).

Between locations, for the same species, the extent of the external water layer (and consequently fractionation) is influenced by wetness and wind. The δ^13^C_C_ data for *C. aciphyllum* suggest that the conditions at the photosynthetic apices of the moss are either windier or drier at ELE than at NOR (or a combination of the two). A similar depletion was measured between the *P. strictum* samples, but with fewer samples the difference was not statistically significant. Meteorological information from ELE is very limited but, between December 1970 and March 1971, the mean wind speed was 26 km h^−1^ (O’Brien 1974) whilst at Palmer station (close to NOR) the mean was 19.5 km h^−1^ (LTER 2014). Whilst these measurements are not comparable in time period, the isolated location of ELE, exposed to the westerly winds at the boundary between Drake Passage and Weddell Sea, and the high altitude of the moss banks (ca. 200 m a.s.l. compared to 20 m a.s.l. at NOR), suggest that higher winds may be a feasible and important factor in determining the microclimate conditions at the moss surface. Thus there is partial support for hypothesis 2, that δ^13^C_C_ is a record of photosynthetic conditions, but any strong latitudinal dependence is overridden by local growing season conditions.

The latitudinal gradient in δ^18^O_C_ was significant when all the samples were pooled, providing further support for hypothesis 1 (that moss water and cellulose δ^18^O values are markers of precipitation composition), but substantial variation remains to be explained. δ^18^O_C_ is not a direct reflection of the isotopic composition of precipitation due to evaporative enrichment, water vapour isotopic exchange, and fractionation during synthesis of isotopic exchange between water and organic molecules, and the temporal offset between precipitation and cellulose synthesis. However, the enrichment factor between cellulose and moss water was greater than the 27 ± 3 ‰ biochemical fractionation factor (Barbour 2007; but see Sternberg and Ellsworth (2011), suggesting that evaporative enrichment occurred in the moss tissue prior to cellulose synthesis. The majority of moss cellulose synthesis takes place within the small, photosynthetic leaf tips, so the evaporative enrichment of the leaf water can be imprinted onto the primary assimilate and subsequently the cellulose. The small volume of water within the photosynthetic moss apices is in contrast to the larger volume of water associated with the non-photosynthetic tissues that comprised the majority of the 50-mm-deep moss samples, and the moss tissue water analysed here. Being lower in volume, and more exposed to wind, the isotopic composition of the water within the apices will change more rapidly and likely undergo more evaporative enrichment than the external tissue water, potentially explaining the lack of tight isotopic coupling between the external water and the cellulose.

### Testate amoebae as palaeoenvironmental proxies on the AP

The dominance of small, cosmopolitan taxa within low diversity, low-concentration testate amoeba assemblages is consistent with previous research in AP (Mieczan and Adamczuk 2014; Smith and Wilkinson 2007; Wilkinson 2001; Yang et al. 2010), Arctic (Beyens et al. 1986, 1990, 1992) and sub-Antarctic (Vincke et al. 2004, 2006) peatlands as well as in *Polytrichum* spp.-dominated temperate peatlands (Mieczan 2009; Mitchell and Gilbert 2004). *M. radiata* type is reported here for the first time from the Antarctic region (Meisterfeld, unpublished data), but its frequent occurrence suggests it is an integral part of the regional fauna that may have been previously overlooked. With the exception of NOR samples dominated by *M. radiata* type (Fig. S1, group 1), the mixed distribution of sites throughout the clusters suggests that living conditions for testate amoeba are relatively homogeneous along the site transect, which is supported by microclimatic data (Fig. S1).

The three indicators of testate amoebae productivity (concentration, estimated biomass and species diversity) tended to covary between sites, with larger populations having higher biomass and diversity. ELE, the northernmost site, had the highest productivity values, suggesting some degree of latitudinal temperature dependence of testate populations, which is supported by the CCA results where latitude was aligned with the primary axis of variability (Fig. 8) and the previously defined latitudinal ‘depauperization’ of Antarctic and sub-Antarctic testate amoeba fauna (Smith 1996). However, GRE had higher concentration, estimated biomass and species diversity than either ARD or NOR, suggesting that any such effect is not straightforward (Table S5). Only when AP productivity data from Signy Island (South Shetland Islands; unpublished) and Lazarev Bay (Alexander Island; Royles et al. 2013a) are included does evidence for gradients in testate productivity indicators become more convincing (Table 1). The similarity of recent summer temperature records from Orcadas (near Signy) north of Rothera (near Lazarev) in the south, alongside the general mixing of sites in the cluster analysis (Fig. S1) suggests that other variables must be driving differences in productivity (Mieczan and Adamczuk 2014; Smith 1996). However, rapid increases in AP testate amoebae concentration over the last 50 years have been linked to the regional warming trend (Royles et al., 2013a), which is plausible because the ca. 3 °C temperature change is far greater than the spatial differences in contemporary climate (Figs. 1, 2). Relatively little research has been undertaken into the long-term effects of warming on testate amoebae populations. In situ [2 years (Jassey et al. 2013)] and growth chamber [8 weeks (Jassey et al. 2013)] experiments demonstrated significant effects of temperature on testate amoebae community structure, but with opposing positive and negative effects on abundance and biomass. In a Sub-arctic *Sphagnum* peatland, summer warming was associated with decreased species richness and community compositional shift towards xerophilous taxa (Tsyganov et al. 2012). On the AP, the recent warming trend has been associated with increased moisture availability and therefore an increase in xerophilous taxa is unlikely in this context. Moisture content was only weakly correlated to CCA axis 2 (Fig. 8), meaning that the limited variability measured across the transect may have been insufficient to drive observable change, although the generally high moisture contents mean that dryness is unlikely to drive the observed low concentrations and diversity (cf. Mieczan and Adamczuk 2014).

The relative homogeneity of testate amoeba community structure and associated environmental data over the 600-km gradient of sites in this study mean that uncertainty remains as to the key drivers of assemblage changes over space and time, meaning that hypothesis 3 can be neither fully accepted nor rejected. Enigmatic variations remain in the dataset, such as the lack of *A. muscorum* at ARD and the much reduced concentrations of *C. dubium* and *M. radiata* type at NOR and ELE/ARD respectively. Whilst these features could result from stochastic variability relating to biogeographical factors (e.g. Yang et al. 2010), the cosmopolitan nature of the taxa involved makes this unlikely and suggests that there are relationships between testate amoebae and their (micro-)environmental setting that remain to be determined.

### Regional versus local factors in driving contemporary AP moss ecosystems

Meteorological observations, along with model outputs of future climate and species distribution, are generated over spatial scales orders of magnitudes larger than individual organisms (Potter et al. 2013). In contrast, for all organisms, and especially for largely sessile microbes and plants, it is the local microclimate conditions which are critical to determining the timing and extent of metabolic activity. For example, whether moss is growing in sun or shade, or within a small cushion or a large moss bank, determines the leaf temperature. Subsequently leaf temperature, largely through enzyme activity, affects net assimilation and testate amoebae division rates, and, consequently, determines the dynamics for organic matter synthesis and microbial population development.

Similarly, there are local impacts that can decouple the isotopic composition of leaf water from the annual average precipitation input. Specifically, the extent of isotopic evaporative enrichment is dependent on the microclimatic sunshine, wind speed and wetness, all individually functions of moss surface microtopography (Lucieer et al. 2013; Ménot-Combes et al. 2002) and the water turnover time. AP summers are wet so, as the high moisture contents of the moss samples suggest, desiccation of moss tissue is rare, particularly over the 5-cm depth sampled here. However, the photosynthetically active upper 5 mm green tips are likely to go through substantially more wetting–drying cycles than the denser, more protected, non-photosynthetic tissue below. The moss water volume will be filled following the influx of precipitation or melt water, whilst available free water is potentially lost to evaporation, percolation, drainage and freezing. Consequently the moss water represents a weighted average of the inputs minus the outputs integrated between freezing events, rather than just reflecting the most recent wetting event.

Moisture content also has a significant influence on the extent of ^13^C discrimination, with mosses in wet patches in the bottom of hollows, or growing in flushes, having long metabolically active periods, but, coupled with restricted CO_2_ diffusion, limited discrimination against ^13^CO_2_ throughout the growing season. Moss shoots growing on the microtopographical peaks experience windier conditions (Wasley et al. 2012), dry out quickly and thus have a short active period, but spend a higher proportion of that active time with little CO_2_ diffusion limitation than those growing in wet flushes. Consequently, as the data here suggest, the drier mosses discriminate more against ^13^CO_2_ and will have organic matter relatively depleted in ^13^C, and this sensitivity is important for the use of these species as palaeoclimate proxies (Bramley-Alves et al. 2015). The surface topography is unlikely to have changed over the course of growth of the contemporary samples and, thus, with climate, species and CO_2_ inputs equal the measured ^13^C values should provide a good reflection of current microtopographical conditions. However, on translation into the palaeo-context this scenario has important implications. Depending upon the relative accumulation rates of mosses in peaks and hollows over time, a surface point could have switched microtopographical position affecting the isotope discrimination measurements within a peat core. There is thus a need for comparison of multiple records from separate locations so that consistency between records can be used to test whether changes have been driven by climate or local change.

### Interpretation of contemporary proxy measurements

Even amongst contemporary ecosystems in an environment with strong physical drivers and little biotic competition, the responses of biological systems are not directly coupled to average climate or instantaneous physical inputs. Rather, they are strongly influenced by microclimate, as the time periods for metabolic activity are limited and generally have non-linear responses to temperature. This dependence on microclimate of all the measured proxies has both benefits and problems for interpreting palaeoclimate archives. Proxy records will rarely be directly interpretable in terms of climate variations recorded at a meteorological station. However, when consistent variations between locations are observed, they are likely to be due to a wide scale climatic signal, whilst perturbations recorded at only one or a sub-set of sites will most likely reflect local effects. In addition, depending upon the resolution of analysis, preserved material provides a temporally integrated signal over periods of a single season to decades. Surface moss tissue water can be analysed to provide an estimate of the annual average precipitation composition in regions without regular monitoring. The isotopic composition of cellulose is a weighted average from the growing season, whilst the testate amoebae population represents the microbial biomass present throughout the moss growth interval, which may encompass several generations within one annual cycle.

### Can moss isotope values and testate amoebae be used as palaeoclimate proxies on the AP?

Substantial temporal variation in δ^13^C_C_ values (Royles et al. 2012, 2013a) and testate amoeba populations (Royles et al. 2013a) have been measured in moss cores from the AP and continental Antarctica (Bramley-Alves et al. 2014; Clarke et al. 2012), whilst δ^18^O and testate amoeba variations over time are recorded in moss-based contexts from around the world. The relative consistency of contemporary measurements of potential proxies shown here means that where temporal variations are determined in future, down-core proxy measurements that cover hundreds or thousands of years we can conclude that the environmental conditions in which they were generated were beyond the range of conditions currently found across our transect of the AP.

## Electronic supplementary material

Below is the link to the electronic supplementary material.

Supplementary material 1 (DOC 254 kb)
