# Supplementary material for: Moss stable isotopes (carbon-13, oxygen-18) and testate amoebae reflect environmental inputs and microclimate along a latitudinal gradient on the Antarctic Peninsula
Source: Oecologia. 2016 Mar 22;181:931–45. doi: 10.1007/s00442-016-3608-3 (PMC4912596; doi:10.1007/s00442-016-3608-3)
Supplement: Supplementary file 1 — Supplementary material 1 (DOC 254 kb) [file 442_2016_3608_MOESM1_ESM.doc]

# Electronic Supplemental Material: Moss stable isotopes (^13^C, ^18^O) and testate amoebae reflect environmental inputs and microclimate along a latitudinal gradient on the Antarctic Peninsula, Jessica Royles (jr328@cam.ac.uk), Matthew J. Amesbury, Thomas P. Roland, Glyn D. Jones, Peter Convey, Howard Griffiths, Dominic A. Hodgson, Dan J. Charman

| **Table S1**: Details of sampling locations and physical characteristics of surface moss tissue  (†= 5% Lichen cover; *= 50% moribund tissue) | | | | | | | | |
| --- | --- | --- | --- | --- | --- | --- | --- | --- |
| Location | Sub-Site | Year | Latitude  (^o^S) | Longitude  (^o^W) | *Chorisodontium aciphyllum*  *(%)* | *Polytrichum strictum*  *(%)* | Moisture Content (%) | Bulk Density (g cm^-3^) |
| Elephant | 1A | 2012 | 61° 08’ 36.1’’ | 054° 42‘ 01.4’’ | 90 | 10 | 63 | 0.099 |
| Elephant | 1B | 2012 | 61° 08’ 36.1’’ | 054° 42‘ 01.4’’ | 10 | 90 | 68 | 0.099 |
| Elephant | 1C | 2012 | 61° 08’ 36.1’’ | 054° 42‘ 01.4’’ | 90 | 5† | 39 | 0.081 |
| Elephant | 1D | 2012 | 61° 08’ 36.1’’ | 054° 42‘ 01.4’’ | 30 | 70 | 65 | 0.091 |
| Elephant | 1E | 2012 | 61° 08’ 36.1’’ | 054° 42‘ 01.4’’ | 90 | 10 | 57 | 0.105 |
| Elephant | 1F | 2012 | 61° 08’ 36.1’’ | 054° 42‘ 01.4’’ | 90 | 10 | 69 | 0.136 |
| Elephant | 1G | 2012 | 61° 08’ 36.1’’ | 054° 42‘ 01.4’’ | 100 | 0 | 44 | 0.122 |
| Elephant | 1H | 2012 | 61° 08’ 36.1’’ | 054° 42‘ 01.4’’ | 0 | 100 | 69 | 0.103 |
| Elephant | 1I | 2012 | 61° 08’ 36.1’’ | 054° 42‘ 01.4’’ | 100 | 0 | 62 | 0.096 |
| Elephant | 1J | 2012 | 61° 08’ 36.1’’ | 054° 42‘ 01.4’’ | 0 | 100 | 69 | 0.114 |
| Elephant | 2A | 2012 | 61° 08’ 26.5’’ | 054° 41‘ 50.0’’ | 80 | 20 | 69 | 0.052 |
| Elephant | 2B | 2012 | 61° 08’ 26.5’’ | 054° 41‘ 50.0’’ | 10 | 90 | 71 | 0.097 |
| Elephant | 2C | 2012 | 61° 08’ 26.5’’ | 054° 41‘ 50.0’’ | 0 | 100 | 75 | 0.081 |
| Elephant | 3A | 2012 | 61° 08’ 27.5’’ | 054° 42‘ 39.8’’ | 50 | 50 | 70 | 0.116 |
| Elephant | 3B | 2012 | 61° 08’ 27.5’’ | 054° 42‘ 39.8’’ | 100 | 0 | 70 | 0.088 |
| Elephant | 3C | 2012 | 61° 08’ 27.5’’ | 054° 42‘ 39.8’’ | 0 | 100 | 74 | 0.135 |
| Elephant | 3D | 2012 | 61° 08’ 27.5’’ | 054° 42‘ 39.8’’ | 50 | 50 | 74 | 0.197 |
| Elephant | 3E | 2012 | 61° 08’ 27.5’’ | 054° 42‘ 39.8’’ | 60 | 40 | 67 | 0.188 |
| Ardley | 1A | 2012 | 62° 12’ 50.1’’ | 058° 55’ 33.4’’ | 100 | 0 | 67 | 0.106 |
| Ardley | 1B | 2012 | 62° 12’ 50.1’’ | 058° 55’ 33.4’’ | 95 | 5 | 76 | 0.095 |
| Ardley | 1C | 2012 | 62° 12’ 50.1’’ | 058° 55’ 33.4’’ | 80 | 20 | 74 | 0.086 |
| Ardley | 1D | 2012 | 62° 12’ 50.1’’ | 058° 55’ 33.4’’ | 100 | 0 | 70 | 0.119 |
| Ardley | 1E | 2012 | 62° 12’ 50.1’’ | 058° 55’ 33.4’’ | 100 | 0 | 75 | 0.11 |
| Ardley | 2A | 2012 | 62° 12’ 47.7’’ | 058° 56’ 30.5’’ | 100 | 0 | 69 | 0.079 |
| Ardley | 2B | 2012 | 62° 12’ 47.7’’ | 058° 56’ 30.5’’ | 20 | 80 | 73 | 0.135 |
| Ardley | 2C | 2012 | 62° 12’ 47.7’’ | 058° 56’ 30.5’’ | 40 | 60 | 71 | 0.11 |
| Ardley | 2D | 2012 | 62° 12’ 47.7’’ | 058° 56’ 30.5’’ | 80 | 20 | 74 | 0.124 |
| Ardley | 2E | 2012 | 62° 12’ 47.7’’ | 058° 56’ 30.5’’ | 20 | 80 | 80 | 0.092 |
| Ardley | 2F | 2012 | 62° 12’ 47.7’’ | 058° 56’ 30.5’’ | 10 | 90 | 79 | 0.097 |
| Norsel | 1A | 2012 | 64° 45’ 33.07’’ | 064° 05‘ 05.4’’ | 20 | 80 | 76 | 0.089 |
| Norsel | 1B | 2012 | 64° 45’ 33.07’’ | 064° 05‘ 05.4’’ | 100 | 0 | 72 | 0.095 |
| Norsel | 1C | 2012 | 64° 45’ 33.07’’ | 064° 05‘ 05.4’’ | 100 | 0 | 75 | 0.121 |
| Norsel | 1D | 2012 | 64° 45’ 33.07’’ | 064° 05‘ 05.4’’ | 50 | 50 | 69 | 0.146 |
| Norsel | 1E | 2012 | 64° 45’ 33.07’’ | 064° 05‘ 05.4’’ | 0 | 100* | 76 | 0.159 |
| Norsel | 1F | 2012 | 64° 45’ 33.07’’ | 064° 05‘ 05.4’’ | 100 | 0 | 71 | 0.114 |
| Norsel | 1G | 2012 | 64° 45’ 33.07’’ | 064° 05‘ 05.4’’ | 0 | 100 | 73 | 0.111 |
| Norsel | 1H | 2012 | 64° 45’ 33.07’’ | 064° 05‘ 05.4’’ | 0 | 100 | 73 | 0.115 |
| Norsel | 1I | 2012 | 64° 45’ 33.07’’ | 064° 05‘ 05.4’’ | 100 | 0 | 71 | 0.104 |
| Norsel | 1J | 2012 | 64° 45’ 33.07’’ | 064° 05‘ 05.4’’ | 100 | 0 | 81 | 0.078 |
| Norsel | 2A | 2012 | 64° 45 ’ 34.9’’ | 064° 04‘ 59.4’’ | 100 | 0 | 75 | 0.104 |
| Norsel | 2B | 2012 | 64° 45 ’ 34.9’’ | 064° 04‘ 59.4’’ | 100 | 0 | 73 | 0.075 |
| Norsel | 2C | 2012 | 64° 45 ’ 34.9’’ | 064° 04‘ 59.4’’ | 100 | 0 | 74 | 0.092 |
| Norsel | 2D | 2012 | 64° 45 ’ 34.9’’ | 064° 04‘ 59.4’’ | 100 | 0 | 75 | 0.139 |
| Norsel | 2E | 2012 | 64° 45 ’ 34.9’’ | 064° 04‘ 59.4’’ | 100 | 0 | 78 | 0.077 |
| Norsel | 3A | 2012 | 64° 45’ 37.7’’ | 064° 05‘ 06.7’’ | 90 | 10 | 68 | 0.122 |
| Norsel | 3B | 2012 | 64° 45’ 37.7’’ | 064° 05‘ 06.7’’ | 0 | 100 | 69 | 0.091 |
| Green | 1 | 2013 | 65° 19' 22.6" | 064° 09'05.3" | 0 | 100 | 73 | 0.114 |
| Green | 2 | 2013 | 65° 19' 22.8" | 064° 09'05.4" | 0 | 100 | 67 | 0.102 |
| Green | 3 | 2013 | 65° 19' 23.1" | 064° 09'05.4" | 0 | 100 | 70 | 0.106 |
| Green | 4 | 2013 | 65° 19' 23.6" | 064° 09'05.7" | 0 | 100 | 72 | 0.126 |
| Green | 5 | 2013 | 65° 19' 23.8" | 064° 09'05.1" | 0 | 100 | 74 | 0.178 |
| Green | 6 | 2013 | 65° 19' 23.3" | 064° 09'04.6" | 0 | 100 | 75 | 0.116 |
| Green | 7 | 2013 | 65° 19' 23.1" | 064° 09'04.6" | 0 | 100 | 72 | 0.069 |
| Green | 8 | 2013 | 65° 19' 22.8" | 064° 09'04.7" | 0 | 100 | 70 | 0.106 |
| Green | 9 | 2013 | 65° 19' 23.0" | 064° 09'03.0" | 30 | 70 | 85 | 0.073 |
| Green | 10 | 2013 | 65° 19' 23.3" | 064° 09'03.4" | 0 | 100 | 71 | 0.102 |
| Green | 11 | 2013 | 65° 19' 23.5" | 064° 09'04.1" | 0 | 100 | 74 | 0.122 |
| Green | 12 | 2013 | 65° 19' 24.4" | 064° 08'54.3" | 0 | 100 | 75 | 0.127 |
| Green | 13 | 2013 | 65° 19' 24.4" | 064° 08'54.6" | 30 | 70 | 85 | 0.078 |
| Green | 14 | 2013 | 65° 19' 24.4" | 064° 08'54.3" | 30 | 70 | 75 | 0.106 |
| Green | 15 | 2013 | 65° 19' 22.5" | 064° 09'05.2" | 100 | 0 | 78 | 0.072 |

| **Table S2:** Antarctic Peninsula locations from which water samples were collected (See Fig 3) | | | |
| --- | --- | --- | --- |
| **Location** | **Year** | **Latitude (^o^S)** | **Longitude (^o^W)** |
| Elephant Island | 2012 | 61° 7' 48" | 55° 7' 12" |
| Ardley Island | 2012 | 62° 12' 0" | 58° 55' 48" |
| Barrientos Island | 2012 2013 | 62° 24' 0" | 59° 43' 48" |
| Deception Island | 2012  2013 | 62° 35' 24" | 60° 39' 0" |
| Brown Bluff | 2013 | 63° 31' 48" | 56° 54' 0" |
| Orne Harbour | 2013 | 64° 37' 12" | 62° 31' 48" |
| Cuverville Island | 2013 | 64° 40' 12" | 62° 37' 48" |
| Danco Island | 2013 | 64° 43' 48" | 62° 37' 12" |
| Norsel Point | 2013 | 64° 46' 12" | 64° 6' 0" |
| Janus Island | 2013 | 64° 46' 48" | 64°6' 02” |
| Neko Harbour | 2013 | 64° 48' 0" | 62° 33' 0" |
| Jougla Point | 2013 | 64° 49' 48" | 63° 30' 0" |
| Pleneau Island | 2013 | 65° 6' 0" | 64° 4' 12" |
| Lemaire Channel | 2012 | 65° 7' 48" | 64° 0' 0" |
| Petermann Island | 2013 | 65° 10' 12" | 64° 10' 12" |
| Green Island | 2013 | 65° 19' 12" | 64° 9' 0" |

| **Table S3:** Summary of testate amoeba results in percentage of total count, concentration and biomass form  (see text and Table S4 for further details) | | | | | | | | | | | | | | | | |
| --- | --- | --- | --- | --- | --- | --- | --- | --- | --- | --- | --- | --- | --- | --- | --- | --- |
|  | |  | ***Assulina muscorum*** | ***Corythion dubium* type** | ***Cryptodifflugia sp.*** | ***Difflugia pristis type*** | ***Euglypha rotunda*** | ***Euglypha tuberculata* type** | ***Hyalosphenia elegans*** | ***Hyalosphenia sp.*** | ***Microcorycia radiata* type** | ***Plagiopyxis labiata*** | ***Trinema lineare* type** | ***Valkanovia elegans*** | **Unknown Type** | **Sample mean** |
| Presence (out of n = 61) | | | 37 | 57 | 3 | 8 | 1 | 4 | 1 | 1 | 40 | 4 | 7 | 17 | 7 | - |
| Proportion of total count (%) | Mean (when present) | | 12.7 | 56.1 | 3.2 | 7.4 | 0.6 | 0.8 | 0.9 | 0.5 | 51.1 | 7.1 | 1.4 | 2.5 | 14.6 | - |
|  | Standard deviation (when present) | | 14.4 | 29.6 | 2.0 | 9.4 | - | 0.2 | - | - | 31.7 | 1.9 | 0.8 | 2.7 | 20.8 | - |
|  | Maximum | | 52.7 | 100.0 | 4.6 | 28.3 | 0.6 | 1.0 | 0.9 | 0.5 | 100.0 | 9.1 | 3.0 | 10.0 | 54 | - |
| Concentration (tests dry g^-1^) | Mean (when present) | | 4400 | 14676 | 3786 | 6299 | 157 | 385 | 124 | 165 | 13147 | 7406 | 633 | 756 | 3612 | **27831** |
|  | Standard deviation (when present) | | 9790 | 21658 | 5215 | 11706 | - | 487 | - | - | 20908 | 5825 | 597 | 1372 | 5768 | 35907 |
|  | Maximum | | 52903 | 115602 | 9797 | 33953 | 157 | 1113 | 124 | 165 | 132111 | 13715 | 1747 | 5878 | 16062 | 211924 |
| Biomass  (μgC g^-1^) | Mean (when present) | | 9.4 | 19.0 | 52.4 | 51.6 | 0.1 | 3.8 | 1.0 | 1.2 | 15.9 | 43.3 | 1.0 | 1.8 | 9.4 | **49.8** |
|  | Standard deviation (when present) | | 15.0 | 28.1 | 72.2 | 96.0 | - | 4.8 | - | - | 25.2 | 34.1 | 0.9 | 3.2 | 15.0 | 97.9 |
|  | Maximum | | 125.3 | 149.7 | 135.7 | 278.3 | 0.1 | 11.0 | 1.0 | 1.2 | 159.3 | 80.2 | 2.8 | 13.9 | 41.9 | 590.2 |

| **Table S4:** Information on biovolume and biomass calculations for testate amoebae. All measurements in micrometres.  R = radius, D = diameter, W = width, L = length, H = height/depth. Values in regular text are taken from our own microscopic measurements; italic values are from the taxonomic literature as stated. | | | | | | | | | |
| --- | --- | --- | --- | --- | --- | --- | --- | --- | --- |
| **Species** | **Geometric shape (Mitchell, 2004)** | **R** | **D** | **W** | **L** | **H** | **Literature source** | **Biovolume (μm^3^)** | **Estimated biomass (μgC g^-1^)** |
| *Assulina muscorum* | Ovoid |  |  | 38 | 48 | *18* |  | 21525 | 0.0024 |
| *Corythion dubium* | Ovoid |  |  | 27 | 41 | *16* |  | 11775 | 0.0013 |
| *Cryptodifflugia* type | Hemispheric | 39 |  |  |  |  |  | 125917 | 0.0139 |
| *Difflugia pristis* | Cylindrical-ovoid |  | 41 |  | 54 |  |  | 74522 | 0.0082 |
| *Euglypha rotunda* type | Cylindrical-ovoid |  | *15* |  | *40* |  |  | 7200 | 0.0008 |
| *Euglypha tuberculata* type | Cylindrical-ovoid |  | *40* |  | *70* |  | [Ogden and Hedley (1980](#_ENREF_37)) | 89600 | 0.0099 |
| *Hyalosphenia elegans* | Ovoid |  |  | 51 | 88 | *25* |  | 75207 | 0.0083 |
| *Hyalosphenia* sp. | Ovoid |  |  | 46 | 89 | *25* |  | 68844 | 0.0076 |
| *Microcorycia radiata* type | Hemispheric | 17 |  |  |  |  |  | 10963 | 0.0012 |
| Plagiopyxis labiata | Cylindrical-ovoid |  | 44 |  | 34 |  |  | 53158 | 0.0058 |
| *Trinema lineare* | Cylindrical-ovoid |  | 22 |  | 37 |  |  | 14416 | 0.0016 |
| *Valkanovia elegans* | Ovoid |  |  | 36 | 49 | *18* | Based on *A. muscorum* | 21468 | 0.0024 |
| Unknown Type | Cylindrical-ovoid |  | 34 |  | 26 |  |  | 23710 | 0.0026 |

| **Table S5:** Summary of CCA results (see text for details). | | | | | |
| --- | --- | --- | --- | --- | --- |
| Axes | 1 | 2 | 3 | 4 | Total inertia |
| Eigenvalue | 0.306 | 0.066 | 0.006 | 0.001 | 1.385 |
| Species-environment correlations | 0.730 | 0.436 | 0.161 | 0.210 |  |
| Cumulative percentage variance | | | | | |
| of species data | 22.1 | 26.8 | 27.3 | 27.4 |  |
| of species-environment relation | 80.7 | 98.0 | 99.7 | 100 |  |

**References**

Cash J, Hopkinson J (1909) The British freshwater Rhizopoda and Heliozoa. Volume II. Ray Scociety, London

Cash J, Hopkinson J (1915) The British freshwater Rhizopoda and Heliozoa. Volume III. The Ray Society, London

Charman DJ, Hendon D, Woodland WA (2000) The identification of peatland testate amoebae (Protozoa: Rhizopoa) in peats. Quaternary Research Association London

Ogden CG, Hedley RH (1980) An atlas of freshwater testate amoebae. Oxford University Press, Oxford

**Figure S1:** Dendrogram showing results of cluster analysis on testate amoeba samples (see text for details). Individual samples are colour coded by moss type as per Fig. 7. Dominant testate amoeba for each group(s) are also shown.
